# Imaging Immune Surveillance of Individual Natural Killer Cells Confined in Microwell Arrays

**DOI:** 10.1371/journal.pone.0015453

**Published:** 2010-11-12

**Authors:** Karolin Guldevall, Bruno Vanherberghen, Thomas Frisk, Johan Hurtig, Athanasia E. Christakou, Otto Manneberg, Sara Lindström, Helene Andersson-Svahn, Martin Wiklund, Björn Önfelt

**Affiliations:** 1 Department of Applied Physics, Royal Institute of Technology, Stockholm, Sweden; 2 Department of Microbiology, Tumor and Cell Biology, Karolinska Institutet, Stockholm, Sweden; 3 Department of Chemistry, University of Washington, Seattle, Washington, United States of America; 4 Department of Environmental Health, Harvard School of Public Health, Boston, Massachusetts, United States America; 5 Department of Biotechnology, Royal Institute of Technology, Stockholm, Sweden; Centre de Recherche Public de la Santé (CRP-Santé), Luxembourg

## Abstract

New markers are constantly emerging that identify smaller and smaller subpopulations of immune cells. However, there is a growing awareness that even within very small populations, there is a marked functional heterogeneity and that measurements at the population level only gives an average estimate of the behaviour of that pool of cells. New techniques to analyze single immune cells over time are needed to overcome this limitation. For that purpose, we have designed and evaluated microwell array systems made from two materials, polydimethylsiloxane (PDMS) and silicon, for high-resolution imaging of individual natural killer (NK) cell responses. Both materials were suitable for short-term studies (<4 hours) but only silicon wells allowed long-term studies (several days). Time-lapse imaging of NK cell cytotoxicity in these microwell arrays revealed that roughly 30% of the target cells died much more rapidly than the rest upon NK cell encounter. This unexpected heterogeneity may reflect either separate mechanisms of killing or different killing efficiency by individual NK cells. Furthermore, we show that high-resolution imaging of inhibitory synapse formation, defined by clustering of MHC class I at the interface between NK and target cells, is possible in these microwells. We conclude that live cell imaging of NK-target cell interactions in multi-well microstructures are possible. The technique enables novel types of assays and allow data collection at a level of resolution not previously obtained. Furthermore, due to the large number of wells that can be simultaneously imaged, new statistical information is obtained that will lead to a better understanding of the function and regulation of the immune system at the single cell level.

## Introduction

Many methods used in cell biology are based on bulk measurements on large cell populations. However, cell populations are heterogeneous as individual cells respond differently to *e.g.* various treatments or during interactions with other cells. By having experimental read-outs based on population averages, detection of rare clones or uncommon events is challenging. Development of novel tools, *e.g.* in microfluidics and computing, has facilitated the possibility to do high-throughput analysis on the single cell level sparking a renewed interest in cellular heterogeneity [1]–[5].

Conventional methods for single-cell analysis include flow cytometry [6], allowing thousands of individual cells per minute to be analyzed according to their size, granularity and fluorescence properties in a wide range of applications, *e.g.* viability, protein expression and localization, gene expression, etc. However, flow cytometry cannot perform dynamic analysis of single cells and most instruments do not allow observation of spatial localization of fluorescence within a cell. Thus, additional methods for analyzing single cells are required. Examples of other techniques for single-cell analysis are: i) laser scanning cytometry which allows imaging and quantitative analysis of individual cells in tissues *in situ* [7]; ii) capillary electrophoresis for efficient separation and sensitive detection of whole cell or subcellular samples [8]; and iii) laser capture microdissection for excising and separating single cells from tissue for further analysis, such as gene expression and protein analysis [9]. The major drawback of the latter two techniques is the low throughput, and for many analyses the techniques above are not well suited.

Another common technique for dynamic single-cell studies is optical microscopy. By imaging one cell at a time optical microscopy enables monitoring of processes such as migration, proliferation, and cell-cell interaction. However, tracking multiple single cells manually over time is difficult since cells easily disappear from the field of view unless imaging is performed with low resolution [10]. Furthermore, the analysis is time-consuming and ardous. To achieve optical screening of large numbers of cells, different array solutions where cells are separated into individual compartments have been employed. Such techniques have successfully been applied to several adherent cell types [11], [12], but have proven more challenging for long-term imaging of motile suspension cells. To trap suspension cells various capturing techniques have been applied; e.g. functionalization of shallow wells' interiors with specific ligands or antibodies [13], [14], physical confinement via lids [15], tight well dimensions [16], [17]. None of these techniques support real long-term studies including cell proliferation and also offer limited possibility to study, e.g. migrational behavior and multiple cell-cell interactions.

Immune cell populations are especially interesting to study on a single-cell level since they feature intrinsic variations in response (*e.g.* to specific antigen) as part of their effector function. This study focuses on NK cells, which are lymphocytes of the innate immune system with both cytotoxic and cytokine-producing effector functions [18]. NK cell-mediated recognition is achieved by formation of an immune synapse, a highly organized and dynamic sub-cellular interface involving intercellular protein-protein interactions, signaling, and leading to downstream effector functions [19]. It was originally described in the late 1990s between T cells and antigen-presenting cells where T-cell receptors interact with major histocompatibility complex (MHC) molecules forming supra-molecular activation clusters (SMACS) [20], [21]. Later a similar structure was described also for NK cells [22].

There are several challenges associated with long-term live cell imaging of immune cell interactions. NK and T cells are highly motile and the time scale for immune synapse formation may vary considerably between individual cells making it difficult to collect data efficiently. Furthermore, it is often of interest to correlate dynamic events with delayed processes occurring several hours after initiation of the cell-cell interactions, *e.g.* T cells undergoing clonal expansion upon activation by their specific antigen.

To address these problems we have designed dense arrays of microwells to spatially confine cells allowing us to follow the dynamics of many single cell events *e.g.*, protein clustering at the NK cell immune synapse or killing in several wells in parallel with laser scanning microscopy (LSM). We have evaluated microwells made from two different materials, polydimethylsiloxane (PDMS) and silicon. PDMS is a soft polymer material, often used for fabrication of microstructures in *e.g.* microfluidics [23] and has the advantages of being both biocompatible [24] and cheap to produce, making it attractive for fabrication of single-use chips. Silicon has a higher fabrication cost which is often compensated for by increased stability, adding the possibility to reuse the chips. Both the PDMS and silicon chips were designed and integrated with holders to make them compatible with high-resolution imaging in standard confocal microscopes. The method presented here can help gain basic understanding of NK cell biology and reveal the details of the cell to cell heterogeneity that is becoming more apparent [25].

## Results

### Preparation of PDMS chips for microscopy

**Figure 1:**
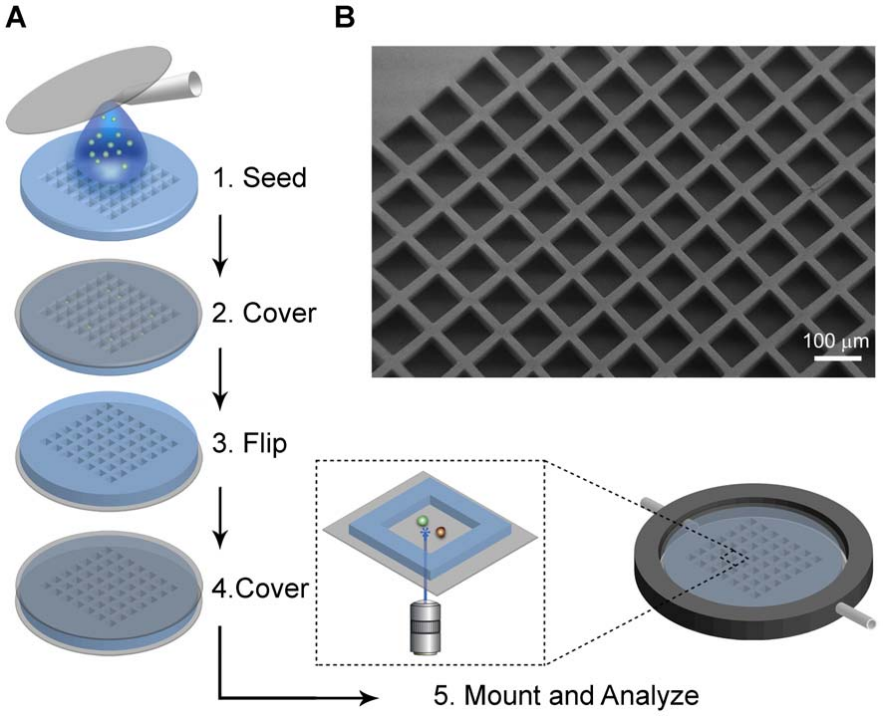
Preparation of PDMS chips for microscopy. (A) Schematic drawing demonstrating chip preparation from seeding to microscopy. Cell suspension was added onto the primed chip (1), excess medium was removed, and the wells were sealed with a cover glass to confine the cells (2). Chip was turned up-side-down (3) and another cover glass was added to increase stability (4). Finally, the chip was mounted in holder and imaging was performed with an inverted microscope to allow high-resolution imaging (5). Illustrations are not to scale. (B) Scanning Electron Microscopy (SEM) image showing the well structure on the chip.

PDMS is an optically transparent soft elastomer with low autofluorescence that can be cast to preferred shape. PDMS multiwell chips were fabricated using a silicon SU-8 mold where the chip was designed to have 100×100 square wells with 80 µm sides and a depth of 45 µm. Untreated PDMS is highly hydrophobic [26], causing medium to be expelled from the shallow wells. To enhance hydrophilicity and facilitate wetting and cell seeding, two different pretreatment techniques were applied; oxygen plasma treatment and fibronectin coating [27], [28]. Both techniques decreased hydrophobicity sufficiently to retain medium within the wells (data not shown). The medium primed chip was seeded by adding cell suspension onto the chip and passively allowing cells to sediment by gravity (Fig. 1A). We found that suspension cells were able to migrate between the shallow 45 µm wells. Additionally, the PDMS was too thick to perform high-resolution imaging. Therefore, we decided to tightly clamp the PDMS chip between two cover glasses and image up-side-down (Fig 1A). In this fashion the wells were sealed against the lower cover glass, similar to a previously described method to select specific antibody-producing B cells [15]. The two cover glasses also increased stability to the PDMS when mounting the chip in a custom made holder. Before imaging the structure of the PDMS chips was analyzed by scanning electron microscopy (SEM) showing that the wells had the desired dimension and quality (Fig. 1B).

### Cell survival in PDMS wells

**Figure 2:**
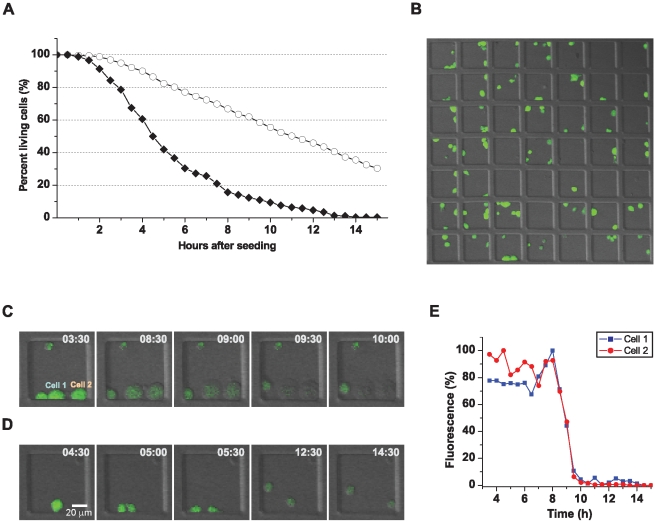
Cell survival in PDMS wells. Calcein-AM-labeled 221 cells were seeded, confined in PDMS wells and imaged at 37°C, 5% CO_2_. Images were acquired of 81 wells every 30 minutes for 15 hours. (A) Cell survival comparing two different techniques to make the wells hydrophilic; fibronectin coating (–○–) and oxygen plasma treatment (–⧫–). The graph shows the weighted values for 5 (plasma treatment) and 7 (fibronectin coating) survival studies. (B) Micrograph of a small part of a fibronectin coated chip that was imaged over time, here showing a snapshot at t = 2 h. (C, D) Time-lapse of two individual wells, showing cell death observed by calcein leakage (C), and a cell dividing during the experiment (D). (E) The calcein intensity of cell 1 and 2 shown in (C) are plotted as percentage fluorescence showing the intensity drop due to leakage of the dye, indicating cell death. Intensity values are compensated for bleaching by normalizing with the intensity of four other cells in the same image that were neither dividing nor dying during the imaging period.

We monitored cell survival over time for the two different surface treatments used to make PDMS hydrophilic. Fibronectin coating resulted in improved survival length compared to plasma treatment (Fig. 2A). Cell survival in the chips was monitored by time-lapse imaging of cells labeled with the vital dye calcein that leaks out of dying cells, scoring the total number of living cells for each time-point. This is exemplified by showing a small part of the chip one hour after seeding (Fig. 2B). Both cell death and proliferation were observed, here shown in time-lapse sequences zoomed in on two individual wells (Figs. 2C–D). Here, cell death was observed as swelling of the cells, suggesting necrosis rather than apoptosis (Fig. 2C). Calcein leaking out of dying cells could be demonstrated by quantifying the percentage of calcein fluorescence over time of the two dying cells (Fig. 2E). At early time points, cell proliferation could be seen for both surface treatments indicating that the cells were healthy at least early on in the experiment.

Increased cell death after oxygen plasma treatment could be due to formation of reactive surface groups. Alternatively, the impaired survival may be due to hydrophobic recovery. It is known that a plasma treated PDMS surface recovers much of its hydrophobicity within a few hours independent of storage conditions [29], [30]. It has been suggested that low-molecular weight polymer chains migrate to the surface to cover up the thermodynamically unstable hydrophilic surface [26]. Despite the limited period of time cells could be followed for both surface treatments, this tool may still be valuable for shorter studies because cells are confined permitting high resolution imaging of dynamic events without the risk of losing track of the cells.

### Preparation of silicon chips for live-cell imaging

**Figure 3:**
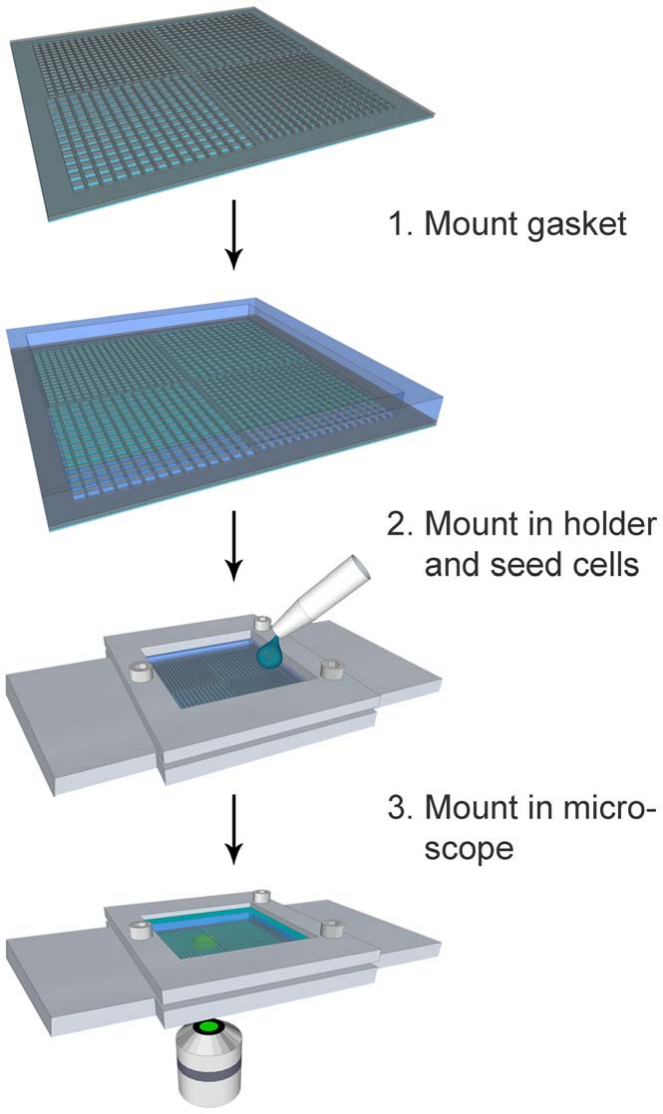
Preparation of silicon chips for microscopy. The silicon chips were primed by degassing the chip submerged in cell medium until the wells were filled with medium. A PDMS-gasket was mounted onto the perimeter of the silicon chip (1). The chip was then mounted in the custom made holder, where tight clamping of the gasket to the chip prevented leakage. Cell suspension was dropped onto the wet chip, and after seeding the chip was washed multiple times (2). Finally, additional medium was added and the chip imaged (3).

Silicon chips were fabricated using anisotropic deep reactive ion etching (DRIE) to achieve 300 µm deep wells with defined geometry. To test for optimal design, a first generation chips were made with four separate areas, each of different well-opening size and distribution. The second generation of chips were fabricated to contain 32 400 wells with 50×50 µm openings placed 30 µm apart, *cf.* Table 1. A reproducible protocol for chip preparation and cell loading was then established (Fig. 3). Prior to seeding of cells, the chip was sterilized and primed with medium. The wet chip was mounted in the holder together with a PDMS gasket to prevent leakage, cell suspension was added, and cells allowed to sediment stochastically into the wells. When the desired cell distribution was reached, the chip was rinsed to remove non-sedimented cells on top of the chip. It is important to note that the wells were deep enough to allow rinsing of these chip without flushing cells from the bottom of the wells.

**Table 1:** Dimensions of first and second generation of chips.

Chip generation	Chip outer dimension (mm^2^)	Well area	Well opening size (µm^2^)	Wall thickness (µm)	No of wells
1^st^	24×24	A	80×80	40	6889
		B	80×80	80	3969
		C	50×50	40	12321
		D	50×50	80	5929
2^nd^	22×22	–	50×50	30	32400

### Cell survival and confinement in silicon wells

**Figure 4:**
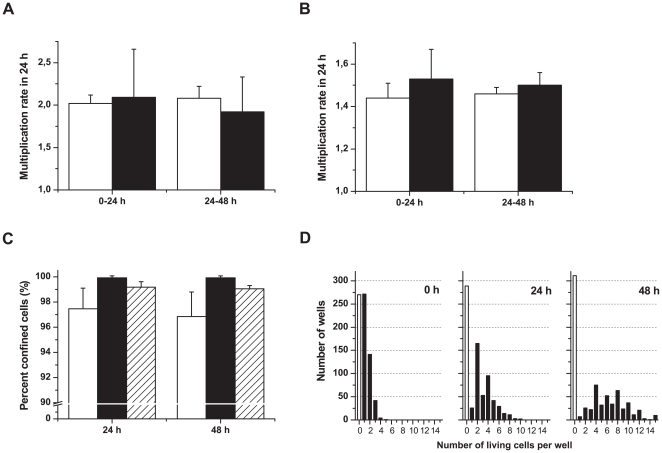
Cell survival and confinement in silicon wells. (A, B) Multiplication rates in the wells (white bars) compared to conventional cell culturing in a tissue culture dish (black bars); 221 cells (A) and YTS cells (B). (C) Level of confinement was measured by quantifying the number of cell-containing wells that had been empty 24 hours earlier and relating it to the total number of empty wells (white bars:221, black bars:YTS, striped bars:293T). The experiments showed a high level of confinement for all three cell types tested. (D) Distribution of 221 cells in the wells over 48 hours after seeding, one representative result out of six is shown. 729 wells were counted in all experiment. The white bar shows the number of empty wells and the slight increase over time is due to cell death in some wells.

Cell culture in silicon microwells was compared to conventional cell culture in tissue culture dishes. We used three different cell lines; the B-cell line 721.221 (221), the NK cell line YTS, and the adherent cell line HEK293T (293T). The multiplication rate in 24 hours was similar in the chip compared to standard tissue culture for both 221 (≈×2), and YTS cells (≈×1.5) (Fig. 4A–B). It was not possible to determine the multiplication rate for the adherent 293T cells due to their tendency to grow up the walls making them difficult to count. Individual wells frequently grew up to higher cell densities (≤15 cells per well) suggesting it is unlikely that the amount of gas exchange or nutrition at the bottom of the wells was restricted.

Next, we wanted to determine whether cells remain confined within the wells they first sedimented into. This is of course of critical importance in long-term assays especially if the cells are not continuously monitored by microscopy. We observed a high degree of cell confinement for all cell types monitored (Fig. 4C). We did observe a small percentage (0.5–2.5% depending on cell type) of initially empty wells containing cells at later time points. A determinative problem was that occasionally cells were trapped halfway down the well during seeding and were thus invisible at experiment start, but detected later after dropping down to the bottom of the well. Furthermore, occasionally we observed that some cells explored the walls of the well and disappeared momentarily out of focus. It was not possible to distinguish this from actual movement of cells between wells, but could be largely reduced by plentiful washing and carefully tapping on the chip after seeding. Since seeding was performed stochastically we optimized the protocol to obtain the largest number of wells containing single cells. This was achieved by seeding approximately 5×10^4^ total cells onto the second generation chips (Fig. 4D), resulting in approximately 1/3 single-cell wells. The proliferation measurements were reproducible for all cell types although the distribution over time had distinct patterns due to different multiplication rates (data not show). The coherent division pattern of the 221 cells, doubling their cell number every 24 hours, was mirrored by preserving more wells with an even number of cells both after 24 hours and 48 hours (Fig. 4D).

The number of empty wells increased slightly over time due to cell death in some wells. It was not possible to reliable count dead cells since cells disintegrated after some time. After 48 hours the cells have lost most of their intracellular dyes probably due to leakage, degradation and dilution when proliferating. In addition, all three cell types tested had a normal morphology for the 48 hours the experiment lasted (data not shown). These results demonstrate that cells are confined and proliferate normally in the silicon wells enabling long-term studies.

### Simulation of cell-cell collision in differently sized wells

**Figure 5:**
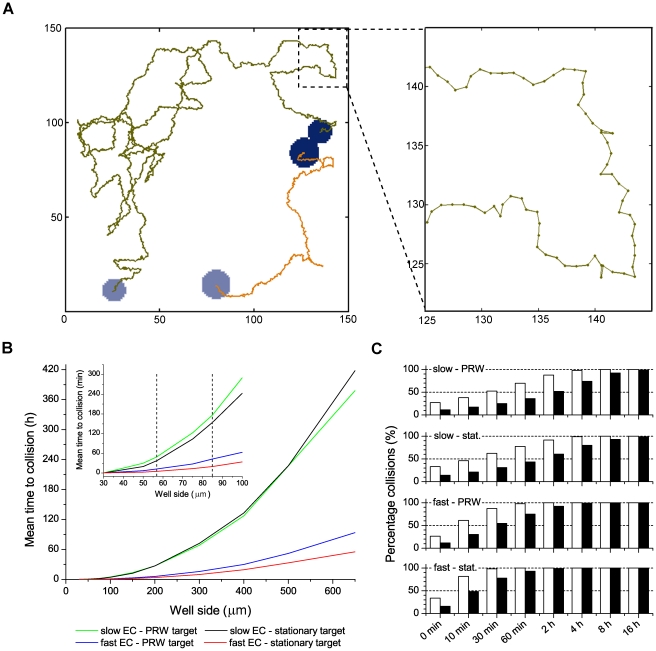
Simulation of time to cell-cell encounter in differently sized wells. One target cell and one effector cell (EC) were randomly placed together in a square well and the mean time to collision was simulated running 1024 times for each well size. The EC was either migrating slow (mean speed = 2 µm/min) or fast (mean speed = 15 µm/min) while the target cell were considered to be either motile (mean speed = 2 µm/min) or stationary. (A) Example of simulated trajectories shown from the time of seeding (light blue circles) to collision (dark blue circles) for a fast EC (Ø 13 µm) with a motile target cell (Ø 16 µm) in a 150 µm well, part of the track is enlarged to show the individual steps. (B) Graph of mean time to collision for the slow EC with migrating (green) or stationary (black) target cell and the fast EC with migrating (blue) or stationary (red) target cell as a function of well side length. Inset shows an enlargement of the graph for smaller well sizes. The two dotted vertical lines correspond to the two well sizes used in the experiments; 57 µm (silicon) and 85 µm (PDMS). (C) Distribution histograms showing the percentage of collisions after a certain time simulated for the two well sizes used in the experiments; white bars:57 µm and black bars:85 µm.

We studied the influence of well size on the expected time to cell-cell collision in square wells of different sizes. In the simulation, one target cell and one effector cell (EC, collective name for NK and T cells), were placed together in a well at a random initial position and the mean time to collision was recorded for 1024 runs for each well size. The target cells were simulated as being either stationary or motile, while the EC was either a “slow” or “fast” EC (cf. below). The cells were considered to move with a persistent random walk [31], [32] as immune cells has been found to walk in consistent directions on the short timescales (min), but exhibit random migration in the long term [33]. The mean speeds used were 2 µm/min for the motile target and slow EC and 15 µm/min for the fast EC. These speeds are selected as they represent extreme values of the mean speeds measured both in vivo and in vitro for NK, B and T cells [34]–[36]. An example of the simulation results is visualized by plotting the trajectories from seeding to collision of a motile target together with a fast EC in a 150 µm well (Fig. 5A).

Well size had a large impact on the mean time to cell-cell collision after seeding (Fig. 5B). The simulations show that it is of limited importance if the target cell is motile or not, the main factor is whether the EC is moving fast or slow. From the same simulated data, distribution histograms of collisions occurring within a defined time period were plotted for the two well sizes used in the experiments, 57 µm and 85 µm (Fig. 5C). With a 57×57 µm^2^ well area, approximately 90% of the fast ECs and more than 50% of the slow ECs had a mean time to collision is in the range of 0–30 minute (Fig. 5C). In contrast, in a 650×650 µm^2^ well the fast ECs on average take 60 hours to meet its target, and for the slow EC this time increases to almost 18 days (Fig. 5B). This clearly demonstrates the benefit of smaller wells for efficient screening of cell-cell interactions unless cells are forced to aggregate, e.g. by using ultrasound [37].

The time to NK and target cell collision was measured to compare our experimental data to the simulated predictions. For these experiments primary human NK cells were mixed with HEK 293T target cells. A second simulation with migration velocity and size of the EC based on data from primary human NK cells, together with a stationary target cell, was performed. The mean time to collision in different sized wells was slightly slower than for the slow EC together with stationary targets (Fig. S1A), possibly reflecting the smaller diameter of the primary NK. We saw a similar collision-time pattern for the simulated NK cells in 57 µm wells (Fig. S1B) and the experimental data (Fig. S1C). For longer incubation times (*ie.* 9 hrs), while the simulations predicated almost all NK cells to have met a target cell, experimentally, only 80% of NK cells were observed to have done so. This difference could be because all simulated cells were migrating permanently while experimentally some NK cells were either temporarily or completely immotile.

### NK cell killing of tumor target cells in microwells

**Figure 6:**
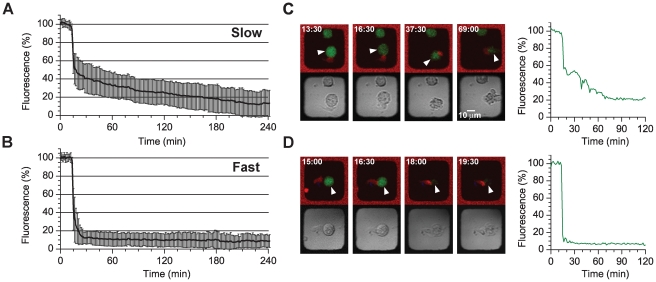
NK cell killing in microwells. IL-2 activated primary human NK cells and 293T target cells were seeded together in a silicon microchip and imaged every 1.5 minutes for several hours. NK cells were stained with calcein red-orange (red) and target cells (arrowheads) with calcein-AM (green) and Far Red DDAO-SE (not shown). (A, B) NK cell induced target cell death was almost always accompanied by a rapid intensity drop of calcein fluorescence. This drop can either be partial, and then followed by slower leakage (A), or almost instantly complete (B). (C–D) Micrographs with the corresponding fluorescence intensity graphs from two different killing events are shown. (C) The NK cell docked with the target cell at 13.5 minutes and immediately initiated killing resulting in an initial 40% calcein intensity drop. At 37.5 minutes an additional 20% had been lost and after 69 minutes only 20% of the calcein remained. This slow leakage was accompanied by blebbing of the target cell. (D) Contact between the NK cell and the target cell was recorded at 15 minutes, followed by an almost immediate intensity drop of 90% at ∼17 minutes.

We wanted to observe NK cell mediated killing of susceptible target cells within the silicon microwells using time-lapse, tile scan imaging to exemplify use of the chip as a tool to rapidly obtain many single cell events. NK cell induced target cell death was detected by monitoring the fluorescence intensity of calcein that leaks out when cells die. Notably, this intensity drop exhibited two distinct patterns; either an initial partial drop followed by slower leakage (Fig. 6A) or an almost instantly complete leakage (Fig. 6B). Fast killing was defined as an uninterrupted initial calcein drop larger than 75% with no or insignificant further leakage while slower killing was defined as a partial initial drop of less than 75% followed by slower leakage. Interestingly, about half of the target cells dying slowly displayed membrane blebbing, while cells with abrupt calcein leaking showed no blebbing but instead rapid swelling and bursting. In total, fast killing made up approximately 30% of all the killing events (n = 117). For clarity, micrographs with the corresponding fluorescence intensity graphs from two individual killing events are shown exemplifying the two observed types of events (Figs. 6C–D). It is possible that these distinct patterns of calcium release could reflect two mechanisms of NK-mediated target cell death or different killing efficiency of individual NK cells. However, there was no correlation between the conjugation time before initial calcein leakage and the type of death induced (data not shown). That NK cells comprise a heterogenous population is becoming increasingly appreciated [10], [25], and indicative of this is that only about 40% of the NK cells making contact with targets display cytotoxicity. Fluorescence screening of NK and target cells confined in microwells greatly facilitated both acquiring and analysis of the data.

### High-throughput high-resolution imaging of immune synapses

**Figure 7:**
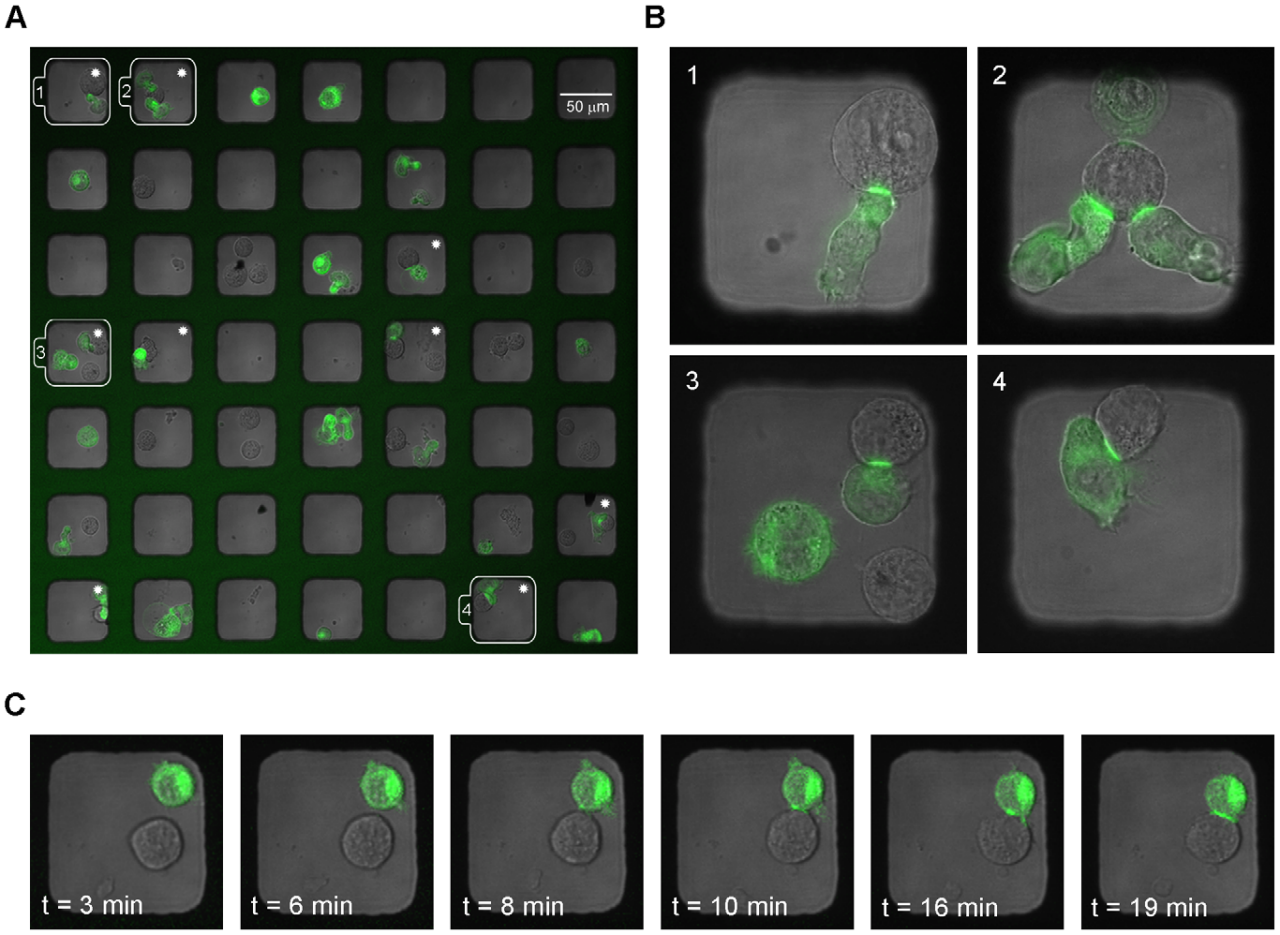
High-resolution imaging of immune synapse formation in microwells. 221/Cw6-GFP target cells (green) were seeded together with the unstained NK cell line YTS/KIR1, and the chip was imaged for synapse formation over time. Inhibitory immune synapse formation can be seen by localized accumulation of HLA-Cw6-GFP at the zone of intercellular contact. (A) Small part of the chip where inhibitory immune synapses are seen in 9 wells (indicated by white stars). After approximately 1 hour, immune synapses could be seen in nearly 20% of the wells shown (9/49), and in 81% (9/11) of the potential wells containing both cell types. (B) Four wells were chosen for high-resolution imaging (63× magnification) as indicated by numbers 1–4. (C) Time-lapse sequence showing the dynamics of synapse formation in a single well containing one NK and one target cell. Here t = 0 refers to the time point when the YTS/KIR1 cell landed on the bottom of the well. Images shown are representative from 4 individual experiments.

The subcellular distribution of specific proteins at the immune synapse is widely believed to determine the outcome of the interaction between NK and target cells. For example, cell surface expression levels of MHC class I have been shown to determine both the protein pattern at the NK cell immune synapse and subsequent cytotoxicity [38]. Additionally, studies of other immune cells similarly suggest that protein patterning at the immune synapse can directly influence downstream signaling [39]. Here, low-resolution microscopy, compatible with rapid screening, was used to identify wells of interest containing both NK (YTS/KIR1) and target cells (221/Cw6-GFP). In a representative image, more than 20% of the wells (11 out of 49) were found to contain both YTS/KIR1 and 221/Cw6-GFP cells after 1 hour of incubation (Fig. 7A). Inhibitory NK cell immune synapses, as defined by HLA-Cw6-GFP clustering at the cell-cell interface between YTS/KIR1 and 221/Cw6-GFP [22], were observed in 9 out of the potential 11 wells containing both target and NK cells. When considering several image windows NK-target cell conjugate formation was observed in 65% of the wells after 1 hour co-incubation (384 out of 592 wells over 3 experiments). The results corresponds well to simulations for the 57 µm wells with slow ECs and motile target cells which predicted about 70% of the cell encounters to occur in less than one hour (Fig. 5C), knowing that not all encounters will result in conjugate formation. Once wells of interest containing NK cell immune synapses were identified they could be imaged by high-resolution 3D laser scanning confocal microscopy (Fig. 7B). It was also possible to perform time-lapse imaging of NK cell immune synapse formation. HLA-Cw6-GFP clustering was initiated almost immediately upon docking and within approximately 10 minutes the intensity maximum at the immune synapse was reached (Fig 7C). These experiments show that this tool has great potential for efficient screening and quantification of the mechanism of immune synapse formation.

## Discussion

Long term live-cell imaging of individual non-adherent cells on glass surfaces is complicated with problems such as drift and cell migration. Here, we have addressed these problems by using chips consisting of arrays of microwells where cells were confined and imaged over time. We evaluate two different systems, one in PDMS and one in silicon, by investigating cell survival. Furthermore, we show examples to illustrate how microwells can facilitate imaging of NK cells as they interact with target cells in activating and inhibitory situations.

Overall, we found the silicon chips to be more practical for our studies compared to the PDMS chips. The sealing procedure used for the PDMS chips proved technically difficult with unreliable cellular distribution across the chip and probably lead to the poor long-term viability observed. This procedure may be avoided by fabricating PDMS wells deep enough to confine cells. However, such structures could pose challenges at fabrication when the delicate structures are to be peeled off the master. Also, with a configuration using deep open wells the remaining layer of PDMS at the bottom of the wells would impede high-resolution imaging. The silicon chips, on the other hand, were easy to handle, gave reproducible distributions of cells, allowed cells to proliferate normally, and were compatible with long-term high-resolution imaging.

The simulation experiments demonstrate the potential benefit in using small wells (30–60 µm^2^) for studies of cell-cell interactions due to the rapid cell-cell collision. The silicon wells used here were 57×57 µm^2^ giving a simulated average time for an exemplified EC to meet its target cell in less than 30 minutes. Thus, even if two cells are confined in a relatively small well it will take some time before the two cells meet, as is also demonstrated in Figure 5. However, for longer incubation times, it is reasonable to assume that two cells in a well have been in contact with each other during the incubation period. This is reflected in our experiments where conjugations are observed in ∼65% of the wells containing both NK cells and target cells approximately one hour after mixing. Furthermore, for the smaller primary NK cells, simulated and experimental data suggest that roughly half of the cells encounter a target cell within 1 hour (Supp. Figs. S1B–C). As some NK cells were found to be temporarily or completely immotile, not all NK cells encountered a target cell in the duration of the experiment. In contrast, all NK cells in the simulation were considered motile, which could account for the difference observed between the simulations and experiments. Taking together factors such as cell seeding and survival and the possibility of doing screening combined with high-resolution imaging our results suggest that wells of ∼50×50 µm^2^ are suitable for studies of interactions between EC and target cells.

The long-term response of individual NK cells following contact with other cells is still largely unknown. For example, it has been shown that human NK cells can kill multiple targets [40], but it is not clear if that leads to late responses such as anergy [41], apoptosis [42] or clonal expansion [43]. With the system presented here we have the possibility to investigate if, for example, the number and quality of target cells seen by an individual NK cell is related to any subsequent responses such as proliferation or apoptosis. Up till now very few studies have been devoted to high through-put studies of immune cells on the single cell level, see for example [44]–[46]. Recently a slightly different approach was described for monitoring of e.g. calcium signaling and target cell killing by CTLs, where individual cells are trapped in small funnel-shaped wells for up to 24 hours [17]. Although this approach seems very useful it does not enable high-resolution imaging, quantification of multiple killing events, migration behavior or studies extending several days. However, similarly to our results for NK cell killing they observed that target cells could be killed either rapidly or more slowly, and propose the possibility of alternate killing mechanisms. Possibly, this overlap reflects processes that are common for NK cells and cytotoxic T cells.

In conclusion, we have shown that silicon microwells are suitable for long-term, parallelized, high-resolution imaging of interactions between single NK cells and target cells. It is becoming increasingly apparent that NK cell repertoires are heterogeneous and that the quality and quantity of an individual's NK cell response depends on the MHC class I background [47], [48]. We believe that the tool presented here will be useful to efficiently assess the heterogeneity of NK cell repertoires, enabling us to address questions related to NK cell education and tolerance.

## Methods

### Chip design and fabrication

Fabrication of the silicon microwells was performed with standard photolithographic processes and metal (first generation) or oxide (second generation) masked DRIE [49]. Following etching, the silicon was oxidized at 1000°C for 24 min to achieve a 200 nm thick SiO_2_ layer. Standard anodic bonding was used to bond the glass to the silicon. The dimensions of the chip was 470 µm thickness in total (wherein 300 µm silicon and 170 µm glass). The first generation chip had a 24×24 mm^2^ outer format and each chip was separated in four different areas (10×10 mm^2^ per area) where the well size and the distances between the wells varied (table 1). The bottoms of the wells were slightly wider than the opening of the wells due to widening during the etching process. True bottom sizes were approximately 83.9±0.7 µm and 55.1±1.1 µm for the first generation chips. A second generation of chips was later fabricated with only one type of wells to increase the density of wells with the preferred well opening size, true size 56.9±0.9 µm. Second generation chips were used for killing studies and immune synapse imaging. Well size, distances and the total number of wells in each area for both types of chips are summarized in Table 1.

PDMS mold grid pattern was drawn in AutoCAD format and transferred to a chrome mask using e-beam (JBX-5DII, JEOL Ltd., Tokyo, Japan) lithography and wet etching in a cleanroom facility. Polished, 100 oriented, unoxidized 3-in. silicon wafers were used as masters for molding. The membrane pattern was transferred to the negative photoresist SU-8 2025 (MicroChem Corp., MA, USA), thus forming a positive relief mold, using a standard UV lithography technique. The resist was developed in XP SU-8. Thickness of the SU-8 structures was confirmed to be 45 µm. For mold release, the master was either silanized in a 1∶10 mixture of dichlorodimethylsilane and trichloroethylene, or surface modified with C4F8 plasma treatment.

Fabrication of PDMS microwells was performed by casting of PDMS and curing agent onto the master, fabricated as described above. The PDMS chip was designed with 100×100 wells sized 80×80 µm^2^ and 45 µm deep. To prepare PDMS for casting, 10 ml Sylgard 184 (Dow Corning, MI, USA) was mixed with 1 ml Sylgard 184 curing agent and stirred for 1 minute. The mixture was degassed in a vacuum dessicator, casted on the master, deposited in a spinner to achieve a thickness of approximately 400 µm, and finally baked for 6 h in an oven at 60°C. Release of the PDMS from the mold was achieved under liquid conditions with ethanol as a lubricant, with consecutive washing in distilled water. After approximately 3 weeks of curing the mechanical properties of the PDMS were stable. The PDMS slab was then cut with a scalpel to match the cover glass. The chip was then bonded to the glass slide by first treating the PDMS surfaces with oxygen plasma from a BD-10ASV spark coil (Electro-Technic Products Inc., IL, USA) for about 30 s.

### Reagents

The fluorescent dyes calcein acetoxymethyl ester (AM) and CellTrace™ calcein red-orange AM (C1430, C34851; Invitrogen, Paisley, Scotland) can be used to determine cell viability in most eukaryotic cells as they are retained in cells with intact plasma membranes but rapidly released from the cell when it dies. CellTrace™ Far Red DDAO-SE (C34553; Invitrogen) is a fixable, far-red fluorescent tracer for long-term cell labeling.

### Cell culture

Two different target cell lines were used for the experiments: 1) The human Epstain Barr Virus (EBV)-transformed B cell line 721.221 referred to as 221, selected to lack endogenous expression of cell surface MHC class I and transfected to express single HLA-Cw6 or HLA-Cw6 coupled to green fluorescent protein (GFP) [50]; 2) the adherent cell line human embryonic kidney 293T (HEK293T) [51]. One NK cell line was used: YTS, both wild type and transfected with the inhibitory receptor KIR2DL1 which recognizes HLA-Cw6. All cell lines were cultivated in supplemented RPMI-1640 (SH30027) with 10% fetal bovine serum (SV30160; Thermo Scientific, MA, USA), 2 mM L-glutamine (G7513), and 100 U/ml Penicillin-Streptomycin (P4333; Sigma Aldrich, St. Louis MO, USA).

Polyclonal primary human NK cells were acquired from lymphocyte-enriched buffy coat residues derived from healthy donors. Peripheral blood mononuclear cells were isolated by centrifugation on Ficoll-Paque Plus gradient (Amersham Biosciences) according to the manufacturer's instructions. NK cells were isolated by negative magnetic bead sorting (Miltenyi Biotec, 130-092-657) according to the manufacturer's instructions. NK cells were cultivated in IMDM supplemented with 10% human serum, 2 mM L-glutamine, 100 U/ml Penicillin-Streptomycin, 1× non-essential amino acids, 1 mM sodium pyruvate and 200 U/ml human Interleukin-2 (IL-2). All cells were kept at 37°C and 5% CO_2_.

### Staining of cells

10^6^ cells were washed twice in RPMI-1640. Staining solution was prepared in 37°C RPMI-1640 and added directly to the pellet for 10 min at 37°C. Final staining concentrations were 0.5 µM for calcein AM, 0.32 µM calcein red-orange or 10 µM Far Red DDAO-SE. Stained cells were washed three times in RPMI-1640 and subsequently used for experiments.

### Microscopy

Images were acquired with a LSM 5 Pascal inverted confocal microscope (Carl Zeiss AG, Göttingen, Germany). A plan neofluar 10×/0.3 objective was used to collect all images except immune synapses, where a plan neofluar 20×/0.5 objective and a plan apochromat 63×/1.4 oil immersion DIC objective were used. The dichroic beam-splitter HFT488/543/633 was used for all fluorescence microscopy, and the individual imaging set up (excitation/filter) for the different fluorescent probes was as follows: calcein AM (488 nm/BP505-530); calcein red-orange (543 nm/BP560-615); and Far Red (633 nm/LP650). To avoid cross-talk between the excitation and emission spectra of different dyes, images were scanned sequentially. Images were analyzed with ImageJ software (US National Institutes of Health, Bethesda, MD, USA). A moving stage enabled automatic collection of images from larger parts of the chip than covered by the sole objectives – series of images were then reconstructed into a mosaic.

### Preparation of PDMS chips for microscopy

The PDMS chip and 30 mm circular cover glasses were sterilized by degassing in 70% ethanol to ensure that the ethanol reached the bottom of the wells. The cleaned chip was fibronectin coated by incubation in a 10 µg/mL fibronectin solution (F0895 from human plasma 1 mg/mL; Sigma-Aldrich, MO, USA) in Dulbecco's phosphate buffered saline (DPBS), for 1h. The chip was rinsed and covered in RPMI-1640. Oxygen plasma treatment was performed as in the bonding procedure described previously but on the well-side of the chip.

Cell survival studies were performed as follows: MOLYKOTE® silicon grease (Dow Corning, MI, USA) was put on the bottom flange of the custom made aluminum holder, the PDMS chip was placed on top and medium containing calcein-AM-labeled 221 cells was added. After cells had settled in the wells, most of the excess medium was removed, a cover glass added, and the sandwich tightly sealed. The holder was then immediately placed in a 37°C, 5% CO_2_ environmental chamber on the microscope. Images were acquired of 81 wells every 30 minutes for 15 hours. Cells were considered alive based on morphology and calcein fluorescence. A square of 9×9 wells was monitored when using the 10×/0.3 objective.

### Preparation of silicon chips for microscopy

Used silicon chips were placed up-side-down in MilliQ water and sonicated for 2×1 hour to remove old cells and debris. Heavily soiled chips were soaked in 5 M NaOH for 10 minutes and then extensively washed in MilliQ. Sterilization was performed by degassing the chip in 70% ethanol to allow the ethanol to reach the bottom of the wells and left for about 30 minutes. To remove all ethanol, chips were submerged in MilliQ for about 3 hours. Next, the chips were primed in RPMI-1640 for 30 minutes and subsequently in supplemented RPMI-1640 for at least 30 minutes before seeding of cells. To load the chip, a PDMS gasket was placed around the perimeter of the well area, the chip mounted in the holder, and cell suspension added. Cells were allowed to sediment under gravity until a distribution of roughly 1–3 cells per well was obtained, with a maximum number of single-cell wells. The chip was then rinsed with medium to remove unwanted cells outside the wells and the chip was left in supplemented RPMI-1640 in the holder.

For cell survival and proliferation studies in the silicon chips, images were obtained from the same 729 wells (3×3 tile scan with 10× objective) every 24 hours after seeding for 3 days. The number of living cells in each well was visually counted from the images, while the control cell numbers in the tissue culture dish were determined using a Bürker chamber. Cells were considered alive based on morphology and calcein fluorescence. Total cell numbers were divided by the total cell number 24 hours earlier to give the multiplication rate. The level of confinement was measured by counting the number of cell-containing wells that had been empty 24 hours earlier; this number was then related to the total number of empty wells 24 hours earlier to calculate the percentage.

### Simulations

All simulations were made using Matlab® (The MathWorks, Natick MA, USA). Four different hypothetic scenarios were simulated; the effector cell (EC) could be fast or slow moving, and the target cells stationary or slow moving. The parameters in the simulation were chosen to cover a large range of the mean migrational speeds immune cells can display at various biological situations. The slow and fast moving ECs were given a mean speed of 2 µm/min and 15 µm/min respectively. Motile cells were simulated as circles, with ECs having a diameter of 13 µm and target cells 16 µm. Stationary cells were simulated as ellipses with a 26 µm major axis and a 15 µm minor axis. All mean cell sizes and mean velocities used were measured directly from imaging of the cell types used in the actual experiments. The simulation was run 1024 times each for a number of different well sizes from 30×30 µm^2^ up to 650×650 µm^2^ and the mean time to collision for each run was recorded. The initial positions of both the target and EC were randomized. If the cells overlapped at the starting positions, the time to collision was set to zero for that run, and the next run started. If a motile cell did not land on top of the other cell, a starting direction was randomly picked. The cell then took 24 1-µm steps during its ‘persistence length’ (thus 24 µm) [52]. During this distance, each new step was taken in a direction picked from a normal distribution with the previous step's direction as the mean, and a standard deviation of π/4. This period of directed movement is due to the fact that a migrating cell adopts a polarity that cannot be abruptly changed [53]. After each persistence length, a new direction was picked from a normal distribution with a standard deviation of π/2 instead. Between each step, the cell would wait for a time taken from a normal distribution with a mean of 30 s for slow cells and 4 s for the fast effector cells, with standard deviations of 15 s and 2 s respectively, which corresponded to the measured means and standard deviations of the speeds. These distributions were truncated at zero so that any negative generated value caused the next step to be taken immediately.

If the cell hit a wall during a step, it would move along the wall for a distance equal to the component of the intended step directed along the wall. The exception to this was the first step of a persistence length, which was picked so that it had no component “into” the wall. This was done to avoid cells reaching a corner staying there for extended periods of time.

A small primary NK cell was also simulated based on experimental data of migrating NK cells together with tumor targets (data not shown). The migration velocity was randomized around 2.3 µm/min with a standard deviation of 0.8 µm/min, the cell was spherically shaped (Ø = 8.3 µm), and all other parameters were set equally as for the slow EC. The target cell was considered to be a stationary sphere (Ø 16 µm).

### NK killing in microwells

The silicon chip was prepared, primary NK cells stained with calcein red-orange and 293T cells stained with calcein AM and Far Red, as described above. To prevent killing before starting the imaging 293T cells were seeded first, then the holder with chip was mounted on the microscope in an environmental chamber at 37°C with 5% CO_2_. A section of the chip was selected and imaged every 1.5 minutes for 9 hours with a plan-neofluar 10×/0.3 objective. NK cells were added after imaging had started and dropped during the first minutes of imaging. Movies were analyzed using the Volocity® software (Improvision, Coventry, UK), to acquire total calcein fluorescence intensity profiles for individual target cells over time. Intensities were normalized to the cell's maximum intensity. The fluorescence intensity profiles were examined manually and classified according these criterions: fast death is an initial uninterrupted loss of >75% fluorescence within a few minutes with no further leakage; slow death shows an initial loss of <75% fluorescence with subsequent slower leakage. Bleaching and was calculated to be responsible for approximately 50% of the intensity loss during experiments lasting 240 min, this estimation is based on target cells in wells with no NK cells (n = 59). The experiment was repeated 4 times with a total of 472 NK cells analyzed. In total the intensity profiles for 32 slow deaths (16 blebbing/16 non-blebbing) and 16 fast deaths were analyzed.

The length of time to collision of NK and target cells was measured from the time when the dropping NK cell became visible to its first contact with a target cell for all wells containing both types of cells. The collision percentage was then calculated based on the total number of NK cells eligible in the individual experiments.

### High-resolution imaging of synapse formation in microwells

A cleaned and primed silicon chip was placed in the holder and approximately 4×10^4^ 221/Cw6-GFP cells were seeded. Once the 221/Cw6-GFP cells had sedimented the chip was mounted on the microscope in an environmental chamber at 37°C with 5% CO_2_. 4×10^4^ unstained YTS/KIR1 cells were added to the chip and imaging started, letting NK cells drop during the first part of imaging. Images were taken with a plan neofluar 20×/0.5 objective every minute for 1 hour. An inhibitory immune synapse formed at the intercellular contact between an NK and non-susceptible target cell is defined by MHC-GFP clustering. Some wells with synapse formation were selected for high-resolution LSCM imaging with a plan apochromat 63×/1.4 oil DIC objective. 3D projections were rendered with ImageJ software (US National Institutes of Health, Bethesda MD, USA) from stacks of approximately 50 *xy*-planes acquired 0.5 µm apart.

## Supporting Information

Figure S1 **Simulation and experimental data of primary NK cells meeting tumor targets in micro wells.** (A–B) In the simulations one EC and one target cell were randomly placed together in a square well and the time to first collision was recorded running 1024 times for each well size. The properties of the EC mimicked that measured for primary NK cells with at diameter of 8.3 µm and migration velocity randomized around 2.3 µm/min while the target cell was considered to be a stationary sphere (Ø 16 µm). (A) Graph of the mean time to collision for all well sizes where inset shows an enlargement of the graph for smaller well sizes, where the two dotted vertical lines correspond to the well sizes used in the experiments; 57 µm (silicon) and 85 µm (PDMS). (B) Distribution histograms of the percentage collisions occurring within a certain time extracted from the same simulation data. (C) Distribution histograms showing the mean percentage of collisions occurring within a certain time measured in experiments of primary NK cells and HEK 293T cells. Presented data is from four individual experiments except for the data point at 9 hours, which only comes from two individual experiments.
