# Supplementary figures and images for: Imaging Immune Surveillance of Individual Natural Killer Cells Confined in Microwell Arrays
Source: PLoS One. 2010 Nov 12;5(11):e15453. doi: 10.1371/journal.pone.0015453 (PMC2980494; doi:10.1371/journal.pone.0015453)

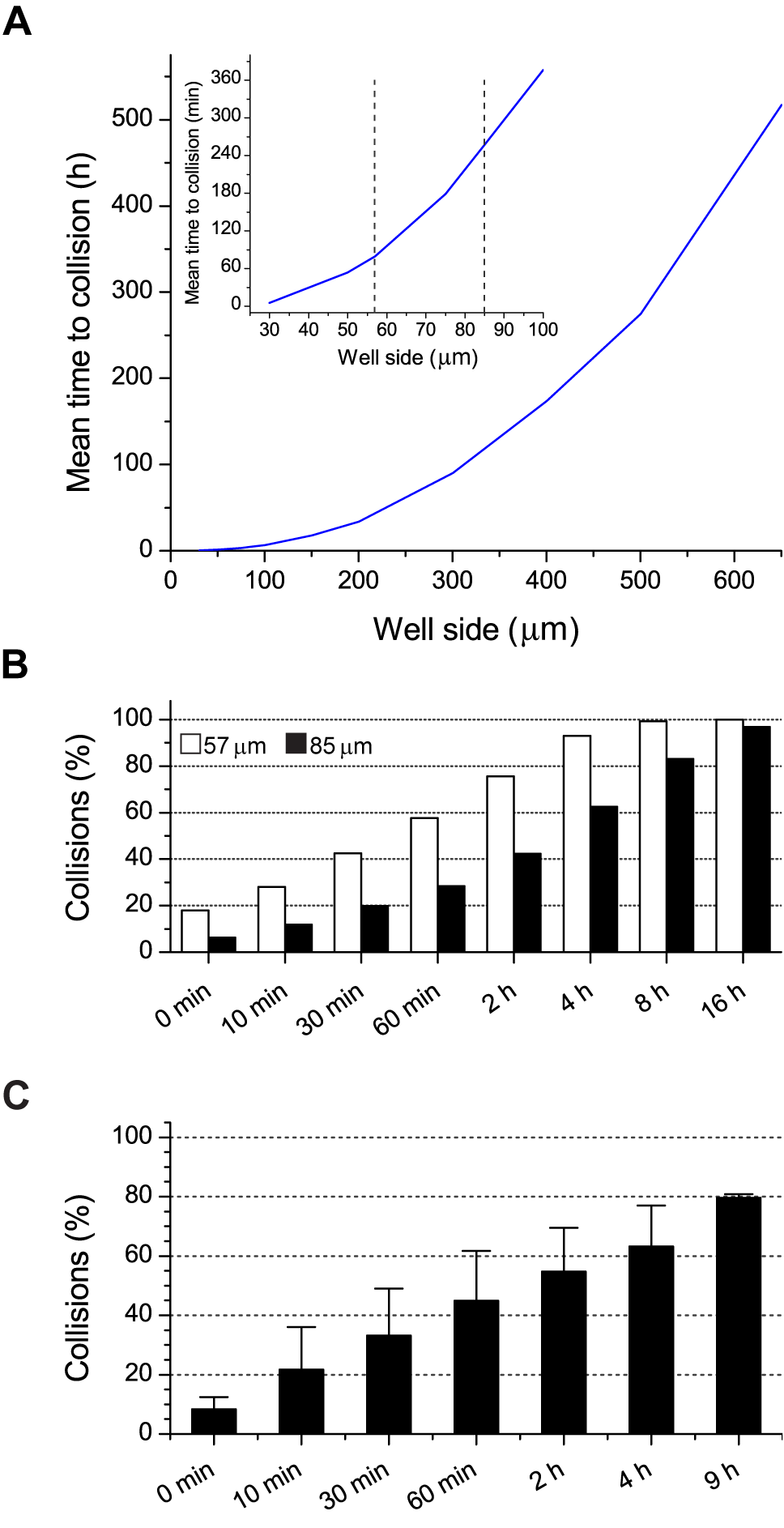

Supplement: Figure S1 — Simulation and experimental data of primary NK cells meeting tumor targets in micro wells. (A–B) In the simulations one EC and one target cell were randomly placed together in a square well and the time to first collision was recorded running 1024 times for each well size. The properties of the EC mimicked that measured for primary NK cells with at diameter of 8.3 µm and migration velocity randomized around 2.3 µm/min while the target cell was considered to be a stationary sphere (Ø 16 µm). (A) Graph of the mean time to collision for all well sizes where inset shows an enlargement of the graph for smaller well sizes, where the two dotted vertical lines correspond to the well sizes used in the experiments; 57 µm (silicon) and 85 µm (PDMS). (B) Distribution histograms of the percentage collisions occurring within a certain time extracted from the same simulation data. (C) Distribution histograms showing the mean percentage of collisions occurring within a certain time measured in experiments of primary NK cells and HEK 293T cells. Presented data is from four individual experiments except for the data point at 9 hours, which only comes from two individual experiments. [file pone.0015453.s001.tif]
